# Association of Daytime Napping with chronic diseases among Tibetan people in China: a cross-sectional study

**DOI:** 10.1186/s12889-021-11871-w

**Published:** 2021-10-08

**Authors:** Wangla Ciren, Qucuo Nima, Yajie Li, Ruifeng He, Deji Suolang, Zhuoga Ciren, Pingcuo Wangqing, Chaonan Fan, Dan Yang, Kunpeng Wu, Meijing Liu, Junmin Zhou

**Affiliations:** 1 Lhasa Chengguan District Center for Disease Control and Prevention, Lhasa, 850000 China; 2 Center for Disease Control and Prevention of Tibet autonomous region, Lhasa, 850000 China; 3 West China School of Public Health and West China Fourth Hospital, Sichuan University, Chengdu, 610041 China

**Keywords:** Tibetans, Napping, Obesity, Hypertension, Diabetes

## Abstract

**Background:**

Obesity, diabetes, and hypertension, as three of the most prevalent chronic diseases, remain a daunting health challenge. However, to our knowledge, no study has made a thorough examination of the association between the three chronic diseases and daytime napping, a widely accepted behavior in many countries. This is especially necessary among Tibetan populations, whose lifestyles and health outcomes may be unique, yet patterns of chronic diseases and napping are under-examined. Thus, we sought to explore the aforementioned association in the Tibetan population of China.

**Methods:**

A total of 2902 participants aged 45–79 in 2019 were included. Multivariate logistic regressions were conducted in 2020. The sex disparity was examined through interaction and stratified analyses.

**Results:**

Hypertension (40.7%) was more prevalent than obesity (20.2%) and diabetes (21.6%). Comparing to non-nappers, those who napped were more likely to have any conditions (OR = 1.30, 95% CI = 1.04–1.62 for 1–59 min/day group and OR = 1.40, 95% CI = 1.10–1.80 for ≥60 min/day group). Participants who had 1–59 min/day of napping were more likely to develop obesity (OR = 1.37, 95% CI = 1.07–1.75), and ≥ 60 min/day of napping was associated with diabetes (OR = 1.33, 95% CI = 1.01–1.74). The interactions between napping and sex were not statistically significant in the models.

**Conclusions:**

The study revealed napping was unfavorably associated with obesity, diabetes, and any conditions in Tibetan people living on the Tibetan Plateau. Future interventions regarding the three chronic diseases may pay more attention to napping.

**Trial registration:**

Not applicable.

**Supplementary Information:**

The online version contains supplementary material available at 10.1186/s12889-021-11871-w.

## Background

Obesity, diabetes, and hypertension, as three of the most prevalent chronic diseases, predispose individuals to cardiovascular diseases (such as stroke and coronary heart disease) and remain a daunting health challenge [1]. Obesity continues to be a major public health challenge across the globe. According to the World Health Organization, 11% of men and 15% of women were obese in 2014 among adults aged 18 years or older [2]. It has resulted in a large fraction of costs not only for healthcare system but also for society [3]. Hypertension is another grave public health concern. The prevalence of hypertension has increased significantly between 1990 and 2015, and the loss of disability-adjusted life-years and deaths associated with hypertension has also increased globally [4]. Diabetes also remains a daunting health threat globally. Its prevalence has significantly increased over the past several decades [5]. The costs associated with diabetes and its consequences are enormous and projected to substantially increase by 2030 [6].

Daytime napping is a widely accepted behavior in many countries. Most people take naps as they believe it is a healthy behavior [7, 8]. A large number of studies have examined its health outcomes. Specifically, although evidence on the association between daytime napping and obesity is inconsistent [9–12], studies have found that daytime napping is significantly related to incidences of diabetes, and others have indicated that it is a risk factor of hypertension and it could lead to metabolic syndrome among various populations [7, 8, 13, 14]. Nevertheless, due to different measurements of napping used (e.g., frequency per week vs. duration per day), different ways of outcomes assessed (e.g., diagnosed diabetes vs. on-site measured diabetes), and different populations investigated, it is difficult to directly compare effects of napping on different chronic diseases from various studies [8]. So, it appears to be necessary to make a thorough examination of the effects of napping on multiple chronic diseases (obesity, diabetes, and hypertension) in a single study.

The Tibetan population is one of the two largest human plateau-dwelling groups globally. Due to the rugged geographical landscape, lack of basic resources and facilities, and low population density, it is difficult to obtain their health-related information [15]. Limited data shows the Tibetan population has unique lifestyles and health outcomes, even comparing with other Chinese populations. For instance, Tibetan people favor butter tea, have very high hemoglobin concentration, and have a low prevalence of diabetes [16, 17]. Nevertheless, this population’s chronic disease status and napping situation remain largely unknown, not to mention their association. Therefore, this study sought to understand daytime napping, chronic diseases (obesity, hypertension, and diabetes), and their association among the Tibetan population of China, using data from the China Multi-Ethnic Cohort (CMEC) Study, a large-scale epidemiological study undertaken in Southwest China, including the Qinghai-Tibet Plateau [18].

In addition, since a number of studies have explored and discussed the sex differences in the risk of chronic diseases [19–21], the study also aimed to explore the sex difference in the association between napping and chronic diseases.

## Materials and methods

### Data

Data in the study comes from the CMEC study, a community population-based study conducted between May 2018 and September 2019 in five provinces (Sichuan, Chongqing, Yunnan, Guizhou, and Tibet) of Southwestern China. A multistage, stratified cluster sampling method was used to obtain samples from Tibetan, Yi, Miao, Bai, Bouyei, Dong, and Han ethnic groups. Firstly, one to two minority settlements for each ethnic group were selected. Secondly, a certain number of communities in each settlement were selected in consideration of the migration status, local health conditions, and ethnic structure. Lastly, all participants who met inclusion criteria (aged 30–79, permanent residents, and willing to complete all requied interviews, physical examinations and blood tests) were invited to participate. Each participant was interviewed with an electronic questionnaire, received medical examinations (such as blood pressure and chest X-ray), and clinical laboratory tests. For clinical laboratory tests, participants provided blood on sites. Venous blood samples, collected after at least 8 h of overnight fasting, were used for clinical laboratory testing including fast blood glucose and hemoglobin A_1c_ (HbA_1c_). The blood samples were temporarily stored at 4 °C before delivered to biobanking or testing by third-party testing laboratories, via cold chain. A detailed description of the CMEC study can be found elsewhere [18]. This study was approved by XXX (information was removed for blinded peer review purpose). Informed consent was obtained from each participant before data collection.

**Fig. 1:**
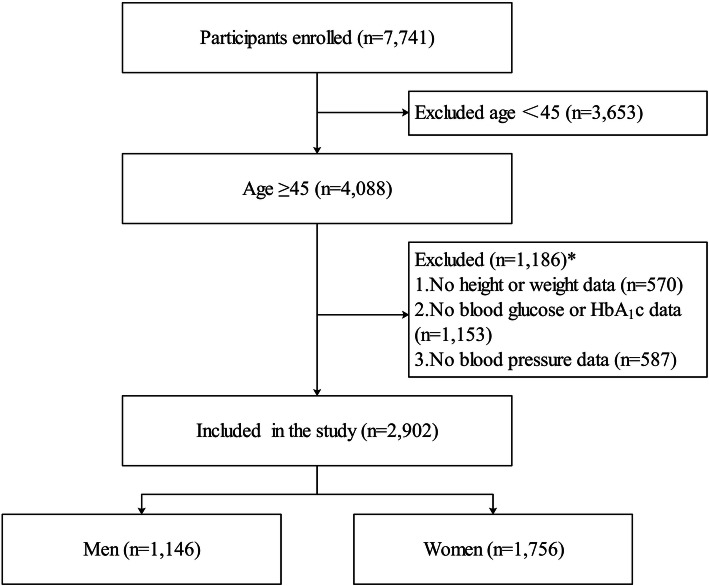
Samples included. Abbreviations: HbA_1c_, Hemoglobin A_1c_ *: Excluded cases may have more than one missing value on height, weight, blood glucose, HbA_1c_, and blood pressure

Data used in the study is from one of the five provinces – Tibet. It was carried out in nine communities (Renqincai village, Luo’ou village, Weiba village, Baiding village, Caigongtang village, Jia’erxi village, Jisu village, Enhuiyuan community, and Luodui community) of Lhasa, Tibet. As shown in Fig. 1, a total of 7741 Tibetan ethnic participants were investigated, and 4839 participants were excluded from the study for the following reasons:

1. Age less than 45;
2. Missing values for height, weight, blood glucose, HbA_1c_, and blood pressure.

The final sample size for the study is 2902, with 1146 men and 1756 women.

Table S1 in the Appendix compared the characteristics of the included sample (*n* = 2902) with the excluded sample due to missing values (*n* = 1186), and no substantial differences were observed between them. Therefore, the exclusion would not pose a grave concern.

### Measurement

#### Obesity

The Chinese BMI criterion was used [22]. Using this scale, underweight is < 18.50, normal weight is 18.50–23.99, overweight is 24.00–27.99, and obesity is 28.00 and over. BMI was calculated by dividing participants’ actual weight in kilograms by the square of height in meters. Height and weight were measured by trained technicians.

#### Hypertension

Measured hypertension: Participants’ blood pressure was measured three times in the physical examinations by using the OMRON HEM-8711 monitor. A participant was divided as having measured hypertension if his or her mean systolic blood pressure was≥140 mmHg and/or mean diastolic blood pressure was≥90 mmHg [23]. Diagnosed hypertension: Participants were asked, “Have you been diagnosed with hypertension by a doctor?” Participants who said “yes” were considered as having diagnosed hypertension. Those who had measured hypertension or/and diagnosed hypertension were defined as having hypertension, otherwise not having hypertension.

#### Diabetes

Measured diabetes: based on the measurements of blood glucose and HbA_1c_, participants were regarded as diabetics if their fasting plasma glucose ≥126 mg/dL and/or HbA_1c_ ≥ 6.5%. The cut-off points were based on recommendations from the American Diabetes Association [24]. Diagnosed diabetes: participants were considered as having diagnosed diabetes if he/she reported “yes” to question “Have you been diagnosed with diabetes by a doctor?”. Those who had measured diabetes or/and diagnosed diabetes were defined as having diabetes, otherwise not having diabetes.

#### Any conditions

Participants were defined as having any conditions if he/she had at least one of the three aforementioned conditions (i.e. obesity, hypertension, and diabetes).

#### Daytime napping

Participants first indicated whether they had a habit of napping. If yes, then they answered the question: “Usually how long (minutes) did you take a nap after lunch?” According to the two questions, participants were grouped into no daytime napping (0 min/day), moderate daytime napping (1–59 min/day), and long daytime napping (≥ 60 min/day) groups.

#### Covariates

Covariates were categorized into four groups: demographics (sex, age, and marital status), socioeconomic gradient (education, annual household income, and employment status), health behaviors (smoking, alcohol consumption, physical activity, nighttime sleep, quality of nighttime sleep), and health-related variable (BMI). Physical activity considered participants’ occupational, traffic, chores, and leisure time activities, and were divided into low and high based on the median value of metabolic equivalent for task (MET). The questions were adapted from validated questionnaires [25, 26], and has been widely used in Chinese [27]. The quality of nighttime sleep was based on the question “In the past 30 days, did you have any sleep problems listed below?”, and options included “It took more than a half-hour to fall asleep, at least three days a week”, “I woke up very early and had been difficult to fall asleep again, at least three days a week”, “I took sleeping pills, at least one day a week”, and “I had difficulty concentrating when working, eating or talking in the daytime because of poor sleep last night, at least three days a week”. If a participant reported at least one of the above, then he/she was regarded as having poor nighttime sleep quality, otherwise having good nighttime sleep quality.

### Statistical analysis

Numbers (proportions) were used to describe variables. Logistic regressions were conducted to examine the relationship between daytime napping and chronic diseases (any conditions, obesity, hypertension, and diabetes) before and after adjusting for potential confounders, including sex, age, marital status, education, annual household income, employment, smoking, alcohol consumption, physical activity, nighttime sleep duration, quality of nighttime sleep and BMI. These potential confounders were selected based on existing literature [7, 12, 28]. We examined the interactions by including a product term for napping and sex in the four multivariate models (any conditions, obesity, hypertension, and diabetes). To explore sex differences in the associations, stratified analyses were carried out. Furthermore, models were run with and without BMI in sensitivity analyses, to test for its possible mediation. Data were analyzed in 2020. The odds ratios (OR) and the corresponding 95% confidence intervals (95% CI) were calculated. SPSS version 21 was used to perform all statistical analyses [29].

## Results

Table 1 shows descriptive statistics for all variables. A total of 2902 participants were included. Among them, 1146 were men and 1756 were women. Overall, the three chronic diseases were prevalent. Any conditions were 58.8% in the sample, 61.3% among men, and 57.2% women. Hypertension (40.7%) was more prevalent than obesity (20.2%) and diabetes (21.6%) in the sample. Sex disparity was observed, as the proportion of obesity was slightly higher in women (21.4%) than in men (18.4%), and those of hypertension and diabetes were higher among men (44.0 and 22.8% respectively) than their female counterparts (38.6 and 20.8% respectively). Notably, the proportion of diagnosed hypertension in all hypertensive participants was low. Specifically, among 1182 hypertension cases, only 804 reported they had been diagnosed before. This was even substantially lower for diabetes. Only 100 out of 630 participants with diabetes reported diagnosis by a doctor.

**Table 1:** Characteristics of Participants in the Sample (*N* = 2902), Men (*n* = 1146) and Women (*n* = 1756)

	Full sample (*N* = 2902) n (%)	Men (*n* = 1146) n (%)	Women (*n* = 1756) n (%)
*Dependent variable*			
Any conditions	1707 (58.8)	702 (61.3)	1005 (57.2)
Obesity	587 (20.2)	211 (18.4)	376 (21.4)
vHypertension	1182 (40.7)	504 (44.0)	678 (38.6)
Diabetes	626 (21.6)	261 (22.8)	365 (20.8)
*Independent variable of interest*			
Daytime napping			
0 min/day	2096 (72.2)	765 (66.8)	1331 (75.8)
1–59 min/day	457 (15.8)	205 (17.9)	252 (14.4)
≥ 60 min/day	349 (12.0)	176 (15.4)	173 (9.9)
*Demographics*			
Age (≥60)	845 (29.1)	351 (30.6)	494 (28.1)
Marital status (cohabited)	2554 (88.0)	1067 (93.1)	1487 (84.7)
*Socioeconomic gradient* Education			
No formal education	1791 (61.7)	623 (54.4)	1168 (66.5)
Elementary school	858 (29.6)	391 (34.1)	467 (26.6)
Middle school and above	253 (8.7)	132 (11.5)	121 (6.9)
Annual household income			
≤ 12,000 CNY	683 (23.5)	227 (19.8)	456 (26.0)
12,000–19,999 CNY	769 (26.5)	297 (25.9)	472 (26.9)
20,000–59,999 CNY	946 (32.6)	395 (34.5)	551 (31.4)
≥ 60,000 CNY	503 (17.3)	227 (19.8)	276 (15.7)
Employed	1976 (68.1)	590 (51.5)	1386 (78.9)
*Health behaviors*			
Smoking			
Never	2254 (77.7)	611 (53.3)	1643 (93.6)
Current	456 (15.7)	363 (31.7)	93 (5.3)
Ever	192 (6.6)	172 (15.0)	20 (1.1)
Alcohol consumption			
No	2117 (73.0)	724 (63.2)	1393 (79.3)
Occasionally (less than once a week)	552 (19.0)	241 (21.0)	311 (17.7)
Frequently (at least once a week)	233 (8.0)	181 (15.8)	52 (3.0)
Physical activity (high, > 17.5 MET-h/day)	1448 (50.0)	513 (44.8)	935 (53.3)
Nighttime sleep (per night)			
< 7 h	194 (6.7)	64 (5.6)	130 (7.4)
7 h^−8^ h	1912 (65.9)	742 (64.8)	1170 (66.6)
> 8 h	790 (27.2)	338 (29.5)	452 (25.7)
Quality of nighttime sleep (poor)	1180 (40.7)	364 (31.8)	816 (46.5)
*Health-related variable*			
Body Mass Index			
Normal	621 (21.4)	258 (22.5)	363 (20.7)
Underweight	34 (1.2)	7 (0.6)	27 (1.5)
Overweight	1660 (57.2)	670 (58.5)	990 (56.4)
Obesity	587 (20.2)	211 (18.4)	376 (21.4)

Abbreviations: CNY, Chinese Yuan Renminbi; MET, Metabolic Equivalent for Task

As for daytime napping, 15.8% of all participants reported 1–59 min napping per day and 12.0% indicated 60 min and more napping time per day. It had been found that men napped more than women (17.9% vs. 14.4% in 1–59 min/day and 15.4% vs. 9.9% in ≥60 min/day, respectively). About 70.9% of participants were middle-aged and the rest were older adults. The educational attainment was low in our sample. Only 8.7% had completed middle school and above education, and the number was much higher in men (11.5%) than in women (6.9%). Men were more likely to report smoking (31.7% for current smokers and 15.0% for ever smokers in men, 5.3% for current smokers and 1.1% for ever smokers in women) and drinking (21.0% drank less than once a week and 15.8% at least once a week in men, 17.7% drank less than once a week and 3.0% at least once a week in women) than women. The physical activity level was not high, as the median value was 17.5 MET-h per day. Most of the participants reported duration of nighttime sleep between 7 and 8 h. Nearly half (40.7%) indicated poor sleep quality, with women (46.5%) suffered more than men (31.8%). The majority of participants were overweight in the sample (57.2%), men (58.5%), and women (56.4%).

Table 2 shows multivariate models assessing the relationship between chronic diseases and daytime napping, controlling for demographics, socioeconomic gradient, health behaviors, and health-related variables. Those who reported daytime napping were more likely to have any conditions (OR = 1.30, 95% CI = 1.04–1.62 for 1–59 min/day group and OR = 1.40, 95% CI = 1.10–1.80 for ≥60 min/day group). Compared with the 0 min/day napping group, participants who had a moderate duration of napping (1–59 min/day) were more likely to be obese (OR = 1.37, 95% CI = 1.07–1.75), and participants who had a prolonged duration of napping (≥ 60 min/day) were more likely to develop diabetes (OR = 1.33, 95% CI = 1.01–1.74). The interactions between napping and sex were not statistically significant in the four models (*P* > 0.05).

**Table 2:** Association between Daytime Napping and Chronic Diseases for All Participants

Variables	Any conditions	Obesity	Hypertension	Diabetes
	Odds Ratio (95% CI)	Odds Ratio (95% CI)	Odds Ratio (95% CI)	Odds Ratio (95% CI)
Crude model				
Daytime napping(/day)				
0 min (ref)	–	–	–	–
1–59 min	**1.41 (1.15, 1.74)**	**1.46 (1.15, 1.84)**	1.21 (0.99, 1.49)	**1.28 (1.00, 1.63)**
≥ 60 min	**1.52 (1.20, 1.93)**	1.27 (0.96, 1.66)	**1.37 (1.09, 1.72)**	**1.40 (1.07, 1.81)**
Multivariate model				
Daytime napping(/day)				
0 min (ref)	–	–	–	–
1–59 min	**1.30 (1.04, 1.62)**	**1.37 (1.07, 1.75)**	1.12 (0.90, 1.39)	1.15 (0.90, 1.47)
≥ 60 min	**1.40 (1.10, 1.80)**	1.27 (0.95, 1.68)	1.20 (0.94, 1.53)	**1.33 (1.01, 1.74)**

Abbreviations: CI, confidence Interval; CNY, Chinese Yuan Renminbi; MET, Metabolic Equivalent for Task

Note: Covariates include: sex, age, marital status, education, annual household income, employment, smoking, alcohol consumption, physical activity, nighttime sleep duration, quality of nighttime sleep, and Body Mass Index (Body Mass Index was not adjusted in the “Any conditions” and “Obesity” regressions)

The results of stratified analyses have been shown in Table S2. The sensitivity analyses (see Table S3 in Appendix) show that odds ratios and 95% confidence intervals did not dramatically change after removing BMI from the models.

## Discussion

The study investigated the association between daytime napping and chronic diseases (obesity, hypertension, diabetes, and the existence of any conditions) using multivariate logistic regressions and examined the sex difference in the relationship by conducting stratified analyses in a Tibetan population aged 45 and above. Our findings suggest that daytime napping was significantly associated with obesity (moderate napping), diabetes (prolonged napping) and any conditions (moderate and prolonged napping) in the full sample. The interactions between napping and sex were not statistically significant in the models.

The prevalence of obesity, hypertension, and diabetes was 20.2, 40.7, and 21.6% respectively. A recent study reported the prevalence of general obesity in Chinese adults was 14.0% [30], which was significantly lower than that in our sample, indicating Tibetans were more likely to have higher BMI compared to other Chinese populations. This was corroborated by our findings that the prevalence of overweight was 57.2%, which is significantly higher than the data (38.8%) from the China Health and Nutrition Survey [31]. According to an existing study, 44·7% of Chinese adults aged 35–75 years had hypertension, which was consistent with our findings [32]. Our findings reveal that 21.6% of participants aged 45 years and above had diabetes, while the data from the China Health and Retirement Longitudinal Study suggests 17.4% in the same age group of the general Chinese population [33]. The prevalence of diabetes in the study also contradicts previous research, which found that Tibetan had lower diabetes prevalence than Han (4.3% vs. 14.7%) [34]. One of the possible explanations could be their participants were aged 18 and above, while ours were greater than 45.

Overall, the prevalence of the three chronic diseases was not low in our study (20.2, 40.7, and 21.6% for obesity, hypertension and diabetes, respectively). This is contrary to our belief that the Tibetan population tends to be healthier than other Chinese. The most likely cause for the discrepancy could be that many cases were undiagnosed, as evidenced by our data (e.g., only 96 out of 626 participants with diabetes in our study reported having been diagnosed by a doctor). In other words, there is a huge health service gap in the population that warrants future research and policy attention.

Of all participants, 27.8% reported napping (either 1–59 min/day or ≥ 60 min/day). This is substantially lower than previous studies mainly concentrating on Han ethnic population [7, 8]. The ethnic disparity might suggest different purposes of napping, which needs future exploration by qualitative research.

The study found that participants who reported napping were more likely to be obese. This could be supported by a previous study which found that napping was positively associated with obesity [12]. It could be explained by existing literature that self-reported napping was significantly related to fat and meat intake [35]. Furthermore, the finding that compared with the 0 min/day napping group, only those who had a moderate duration (1–59 min/day) had higher odds of being obese is different from previous research, which found a significant dose-response association between napping and obesity [12]. This could be due to that only a small portion of participants (12.0%) reported longer duration of napping (≥ 60 min/day) in our study. Further research based on longitudinal data with large sample size is warranted to verify such findings. The biological mechanims underlying the association between napping and obesity has been discussed in previous research, especially in women [12]. Briefly, napping is associated with depression [36], and depression is linked with menopause [37], while the association between menopause and obesity has been found [38]. Therefore, the menopause may have played an important role between napping and obesity.

The relationship between napping and hypertension remained to be insignificant in the sample. Since the findings revealed the significant association between napping and obesity, and obesity is a risk factor for hypertension [39], BMI might have been serving as a mediator between daytime napping and hypertension.

The study demonstrated some evidence of a trend towards the association of napping with diabetes. This is partially in line with an existing study that found that daytime napping is associated with the prevalence of diabetes in women, but not in men [21]. The reason that we did not detect sex difference could be due to the different ethnic groups we studied (Tibetan vs. Han). Alternatively, it could be explained by “the potential type II error” indicated in that study. Specifically, the insignificant association between napping and diabetes in men of that study could result from the relatively small sample size in men (2443 men and 6178 women). The potential mechanism underlying the association has been proposed [7]. Specifically, the circadian oscillator of β cells controls insulin secretion and glucose homeostasis. Prolonged daytime napping (e.g. ≥ 60 min/day) may disturb the circadian rhythm system, and further impact the elevated hemoglobin A1c levels and high homeostasis model assessment of insulin resistance index.

The study has several limitations worth mentioning. First, qualitative data was not collected, so some questions like the purpose of napping could not be answered. Second, the data we used is cross-sectional, thus cannot allow one to infer causality. However, this is a cohort study, and participants will be continuously monitored in the future. Third, the use of self-report data for some variables can introduce recall bias. For instance, the duration of daytime napping was self-reported and thus may not be exactly accurate, as evidenced by the heaping of answers on 30-min intervals. Fourth, although it is clinically accepted [40] and widely-used in research studies [41–43], the OMRON HEM-8711 monitor has not been formally evaluated and validated, which might cause a potential bias in blood pressure measurement.

Despite these limitations, our study is unique. First, our participants were all Tibetans, whose lifestyles and health conditions may be unique to other Chinese populations, yet remarkably little is known on these topics. Our study adds to the extant body of literature by providing evidence regarding their napping habit and chronic diseases. Second, objective measurements were obtained for several key variables. For example, data for obesity, hypertension, and diabetes had relied on medical examinations and clinical laboratory tests (such as blood pressure and HbA_1c_) in addition to self-report. This is especially important, considering many adults with chronic conditions remain undiagnosed [44, 45]. Third, perhaps our study is the first to study the association between napping and the three chronic diseases (obesity, hypertension, and diabetes) in a single study, which could provide references for future research.

## Conclusions

The study established the association of daytime napping with obesity, diabetes, and at least one of the three chronic diseases (obesity, hypertension, and diabetes) in a Tibetan population. Longitudinal studies are needed to be carried out to verify these findings and explore how health interventions could be developed to inform people whether to have daytime napping and if so for how long.

## Supplementary Information

**Additional file 1.**

## Data Availability

The datasets analysed during the current study are not publicly available due to some sensitive information, but are available from the corresponding author on reasonable request.

## Abbreviations

CMEC: China Multi-Ethnic Cohort
HbA_1c_: Hemoglobin A_1c_
BMI: Body Mass Index
MET: Metabolic Equivalent for Task
OR: Odds Ratio
CI: Confidence Interval
