# Supplementary material for: Association of Daytime Napping with chronic diseases among Tibetan people in China: a cross-sectional study
Source: BMC Public Health. 2021 Oct 8;21:1810. doi: 10.1186/s12889-021-11871-w (PMC8501682; doi:10.1186/s12889-021-11871-w)
Supplement: Supplementary file 1 — Additional file 1. [file 12889_2021_11871_MOESM1_ESM.docx]

**Title:** Association of Daytime Napping with Chronic Diseases among Tibetan people in China: A Cross-Sectional Study

Wangla Ciren, MBBS^1,*^; Qucuo Nima, MD^2,*^; Yajie Li, MBBS^2^; Ruifeng He, MBBS^2^; Deji Suolang, MBBS^2^; Zhuoga Ciren, MBBS^2^; Pingcuo Wangqing, AA^2^; Chaonan Fan, MBBS^3^; Dan Yang, MBBS^3^; Kunpeng Wu, BS^3^; Meijing Liu, MBBS^3^; Junmin Zhou, PhD^3^

1 Lhasa Chengguan District Center for Disease Control and Prevention, Lhasa 850000, China.

2 Center for Disease Control and Prevention of Tibet autonomous region, Lhasa 850000, China.

3 West China School of Public Health and West China Fourth Hospital, Sichuan University, Chengdu 610041, China.

* These authors contributed equally to this work and share first authorship.

Correspondence: Junmin Zhou, West China School of Public Health and West China Fourth Hospital, Sichuan University, Chengdu 610041, China. Tel: +86(028)85501548. Fax: +86(028)85501548. Email: junmin.zhou@scu.edu.cn

**Appendix**

Table S1 Comparison of Samples Included (N=2902) and Excluded (N=1186) due to Missing Values

|  | Sample included  n (%) | Sample excluded  n (%) |
| --- | --- | --- |
| *Independent variable of interest* |  |  |
| Daytime napping |  |  |
| 0 min/day | 2096 (72.2%) | 893 (75.3%) |
| 1-59 min/day | 457 (15.8%) | 1519 (12.7%) |
| ≥ 60 min/day | 349 (12.0%) | 140 (11.8%) |
| *Demographics* |  |  |
| Sex |  |  |
| Men | 1146 (39.5%) | 519 (43.8%) |
| Women | 1756 (60.5%) | 665 (56.1%) |
| Age |  |  |
| 45-59 | 2057 (70.9%) | 834 (70.3%) |
| ≥60 | 845 (29.1%) | 352 (29.7%) |
| Marital status |  |  |
| Cohabited (married or not) | 2554 (88.0%) | 1019 (85.9%) |
| Did not cohabit (Separated/divorced/widowed and never married) | 348 (12.0%) | 165 (13.9%) |
| *Socioeconomic gradient*  Education |  |  |
|  |  |  |
| No formal education | 1791 (61.7%) | 718 (60.5%) |
| Elementary school | 858 (29.6%) | 386 (32.6%) |
| Middle school and above | 253 (8.7%) | 80 (6.8%) |
| Annual household income |  |  |
| ≤ 12000 CNY | 683 (23.5%) | 358 (30.2%) |
| 12000-19999 CNY | 769 (26.5%) | 375 (31.6%) |
| 20000-59999 CNY | 946 (32.6%) | 335 (28.3%) |
| ≥ 60000 CNY | 503 (17.3%) | 114 (9.6%) |
| Employment |  |  |
| Employed | 1976 (68.1%) | 788 (66.4%) |
| Unemployed | 925 (31.9%) | 395 (33.3%) |
| *Health behaviors* |  |  |
| Smoking |  |  |
| Never | 2254 (77.7%) | 847 (71.4%) |
| Current | 456 (15.7%) | 262 (22.1%) |
| Ever | 192 (6.6%) | 75 (6.3%) |
| Alcohol consumption |  |  |
| No | 2117 (73.0%) | 845 (71.3%) |
| Occasionally (less than once a week) | 552 (19.0%) | 221 (18.6%) |
| Frequently (at least once a week) | 233 (8.0%) | 118 (10.0%) |
| Physical activity |  |  |
| Low (≤ 17.5 MET-h/day) | 1448 (50.0%) | 591 (49.8%) |
| High (˃ 17.5 MET-h/day) | 1448 (50.0%) | 591 (49.8%) |
| Nighttime sleep (per night) |  |  |
| < 7 h | 194 (6.7%) | 75 (6.3%) |
| 7 h-8 h | 1912 (65.9%) | 771 (65.0%) |
| ˃ 8 h | 790 (27.2%) | 335 (28.3%) |
| Quality of nighttime sleep |  |  |
| Good | 1722 (59.3%) | 790 (66.6%) |
| Poor | 1180 (40.7%) | 394 (33.2%) |

Abbreviations: CNY, Chinese Yuan Renminbi; MET, Metabolic Equivalent for Task

Table S2 Multivariate Logistic Regression on Daytime Napping and Chronic Diseases (Stratified by Sex)

| Daytime napping（/day） | Any conditions | Obesity | Hypertension | Diabetes |
| --- | --- | --- | --- | --- |
|  | Odds Ratio (95% CI) | Odds Ratio (95% CI) | Odds Ratio (95% CI) | Odds Ratio (95% CI) |
| All participants |  |  |  |  |
| 0 min (ref) | - | - | - | - |
| 1-59 min | **1.30 (1.04, 1.62)** | **1.37 (1.07, 1.75)** | 1.12 (0.90, 1.39) | 1.15 (0.90, 1.47) |
| ≥ 60 min | **1.40 (1.10, 1.80)** | 1.27 (0.95, 1.68) | 1.20 (0.94, 1.53) | **1.33 (1.01, 1.74)** |
| Men |  |  |  |  |
| 0 min (ref) | - | - | - | - |
| 1-59 min | 1.38 (0.98, 1.96) | 1.31 (0.87, 1.95) | 1.31 (0.94, 1.84) | 1.30 (0.89, 1.88) |
| ≥ 60 min | 1.36 (0.95, 1.96) | 1.36 (0.89, 2.04) | 1.04 (0.73, 1.49) | **1.58 (1.07, 2.30)** |
| Women |  |  |  |  |
| 0 min (ref) | - | - | - | - |
| 1-59 min | 1.24 (0.93, 1.65) | **1.42 (1.03, 1.94)** | 0.98 (0.73, 1.31) | 1.07 (0.76, 1.49) |
| ≥ 60 min | 1.38 (0.98, 1.96) | 1.14 (0.76, 1.67) | 1.38 (0.98, 1.93) | 1.10 (0.74, 1.63) |

Abbreviations: CI, confidence Interval.

Note: Covariates include: sex, age, marital status, education, annual household income, employment, smoking, alcohol consumption, physical activity, nighttime sleep duration, quality of nighttime sleep, and Body Mass Index (Body Mass Index was not adjusted in the “Any conditions” and “Obesity” regressions).

Table S3 Association between Daytime Napping and Chronic Diseases (Sensitivity Analysis)

| Daytime napping（/day） | Hypertension (Model 1) | Hypertension (Model 2) | Diabetes (Model 1) | Diabetes (Model 2) |
| --- | --- | --- | --- | --- |
|  | Odds Ratio (95% CI) | Odds Ratio (95% CI) | Odds Ratio (95% CI) | Odds Ratio (95% CI) |
| Full sample |  |  |  |  |
| 0 min (ref) | - | - | - | - |
| 1-59 min | 1.12 (0.90, 1.39) | 1.15 (0.93, 1.43) | 1.15 (0.90, 1.47) | 1.20 (0.93, 1.52) |
| ≥ 60 min | 1.20 (0.94, 1.53) | 1.24 (0.98, 1.58) | **1.33 (1.01, 1.74)** | **1.37 (1.05, 1.80)** |
| Men |  |  |  |  |
| 0 min (ref) | - | - | - | - |
| 1-59 min | 1.32 (0.94,1.84) | 1.34 (0.97,1.87) | 1.30 (0.89, 1.88) | 1.35 (0.93, 1.93) |
| ≥ 60 min | 1.04 (0.73,1.48) | 1.07 (0.75,1.52) | **1.58 (1.07, 2.30)** | **1.60 (1.09, 2.33)** |
| Women |  |  |  |  |
| 0 min (ref) | - | - | - | - |
| 1-59 min | 0.98 (0.73,1.31) | 1.01 (0.75,1.34) | 1.06 (0.75, 1.48) | 1.10 (0.78, 1.53) |
| ≥ 60 min | 1.38 (0.98,1.93) | **1.42 (1.01,1.98)** | 1.12 (0.75, 1.65) | 1.17 (0.79, 1.72) |

Abbreviations: CI, confidence Interval.

Note: Model 1 adjusted for: sex, age, marital status, education, annual household income, employment, smoking, alcohol consumption, physical activity, nighttime sleep duration, quality of nighttime sleep, and Body Mass Index; Model 2 removed Body Mass Index from the covariates.
